# The Impact of a Maternal Education Program Through Text Messaging in Rural China: Cluster Randomized Controlled Trial

**DOI:** 10.2196/11213

**Published:** 2018-12-19

**Authors:** Ri-Hua Xie, Hongzhuan Tan, Monica Taljaard, Yan Liao, Daniel Krewski, Qingfeng Du, Shi Wu Wen

**Affiliations:** 1 Department of Nursing Nanhai Hospital Southern Medical University Foshan China; 2 General Practice Center Nanhai Hospital Southern Medical University Foshan China; 3 McLaughlin Centre for Population Health Risk Assessment Faculty of Medicine University of Ottawa Ottawa, ON Canada; 4 Xiangya School of Public Health Central South University Changsha China; 5 Clinical Epidemiology Program Ottawa Hospital Research Institute University of Ottawa Ottawa, ON Canada; 6 School of Epidemiology and Public Health Faculty of Medicine University of Ottawa Ottawa, ON Canada; 7 Obstetrics Maternal Newborn Investigation Research Group Department of Obstetrics & Gynecology University of Ottawa Ottawa, ON Canada

**Keywords:** maternal education, text messaging, maternal health, infant health, cluster trial

## Abstract

**Background:**

In recent years, attempts have been made to use mobile phone text messaging (short message service, SMS) to achieve positive results for a range of health issues. Reports on the impact of maternal education programs based on this widely available, inexpensive, and instant communication tool are sparse.

**Objective:**

This study aimed to explore the impact of a maternal education program through text messaging.

**Methods:**

We conducted a cluster randomized trial in a remote region in the Chinese province of Hunan between October 1, 2011, and December 31, 2012. We used county as the unit of randomization (a total of 10 counties), with half of the counties randomly allocated to the intervention arm (with maternal education material adapted from the World Health Organization being delivered by text messaging to village health workers and pregnant women alike) and the other half to the control arm (normal care without text messaging). Data on maternal and infant health outcomes and health behaviors were collected and compared between the 2 arms, with maternal and perinatal mortality as the primary outcomes.

**Results:**

A total of 13,937 pregnant women completed the follow-up and were included in the final analysis. Among them, 6771 were allocated to the intervention arm and 6966 were allocated to the control arm. At the county level, the mean (SD) of maternal mortality and perinatal mortality rate were 0.0% (0.1) and 1.3% (0.6), respectively, in the intervention arm and 0.1% (0.2) and 1.5% (0.4), respectively, in the control arm. However, these differences were not statistically significant. At the individual level, there were 3 maternal deaths (0.04%) and 84 perinatal deaths (1.24%) in the intervention arm and 6 maternal deaths (0.09%) and 101 perinatal deaths (1.45%) in the control arm. However, the differences were again not statistically significant.

**Conclusions:**

Adequate resources should be secured to launch large-scale cluster randomized trials with smaller cluster units and more intensive implementation to confirm the benefits of the text messaging–based maternal education program suggested by this trial.

**Trial Registration:**

ClinicalTrials.gov NCT01775150; https://clinicaltrials.gov/ct2/show/NCT01775150 (Archived by WebCite at http://www.webcitation.org/74cHmUexo)

## Introduction

### Background

Although maternal and infant death rates in China are not as high as those in some developing countries [1], they are still very high, about 300 per 100,000 deliveries for maternal mortality and 40 per 1000 births for infant mortality in remote areas. Many of these maternal and infant deaths may be avoidable if mothers/local health workers can learn how to better detect and manage pregnancy complications. The World Health Organization (WHO)’s Promotion of Perinatal Care Program [2] contains teaching aids and texts for maternity care education. These WHO materials have been validated and widely implemented in many regions worldwide, with varying levels of success.

In recent years, attempts have been made to use mobile phone text messaging (short message service, SMS) to achieve positive results for a range of health issues including treatment management and adherence [3-5], quality of life and well-being assessment [6], weight management [7,8], suicide prevention [9], smoking cessation [10], and other public health issues [11,12]. Attempts have also been made to use mobile phone text messaging to address issues related to maternal and child health, including interventions to support postabortion contraception [13], infertility treatment [14], prevention of mother-to-child HIV transmission [15], pregnancy nutrition intervention [16], management of gestational diabetes [17], adherence to postpartum care [18], lactation management [19], and infant feeding [20]. Although the overarching goal of mobile phone text messaging seeks to promote behavioral changes in both health care providers and the target population of interest, text messaging interventions evaluated to date have met with varying degrees of success [11,12].

Reports on the impact of maternal education programs based on this widely available, inexpensive, and instant communication tool in low- and middle-income countries are sparse. To our knowledge, none of the studies have yet tried to integrate WHO’s maternity care education material with text messaging as a local maternal and infant health promotion tool in remote rural areas in China. In a systematic review, Amoakoh-Coleman et al identified 19 studies (10 intervention studies and 9 descriptive studies) of mobile health (mHealth) on various maternal and child health issues; they found that none of these studies attempted to integrate the WHO education materials in the education program and none of these studies directly assessed the effect of mHealth on maternal and neonatal mortality [21]. Mobile phones are popular in China (more than 50% of the population has a mobile phone) and accessible (with wireless networks spanning most remote areas), and mobile phone text messaging is affordable (<5 cents per message), making China an opportune place to implement a large-scale maternal education program using this text messaging–based health communication tool.

### Objective

To obtain the empirical data needed to explore this cost-effective novel health promotion opportunity, we designed a cluster randomized trial to evaluate the potential benefits of implementing the WHO maternal education program using text messaging in a remote area in China. We chose a cluster randomized trial because it could be implemented in large scale at a lower cost and it might help to reduce contamination [22].

## Methods

### Ethical Approval, Trial Registration, and Reporting

We obtained approval from the Ottawa Hospital Research Ethics and Confidentiality Committee before commencing the proposed study (REB # 2011467-01H). We registered this trial in the ClinicalTrials.gov Protocol Registration System (registration number: NCT01775150). We followed the Consolidated Standards of Reporting Trials 2010 statement: extension to cluster randomized trials [23] in the reporting of the trial.

### Study Region

This study was conducted in the northwestern region of the Chinese province of Hunan, a mountainous area comprising about 5 million residents. Basic maternity care in this area is provided by village health workers. There are several unique features that make the northwestern region of Hunan province the ideal location for a cluster randomized trial to evaluate the impact of a maternal health education program. First, the authority governing the whole region agrees to participate in this study, so that no further negotiation with local authorities is needed. As a result, possible bias introduced by selective participation is reduced [22]. Second, the region is quite homogeneous, thereby increasing the chance of obtaining a balanced randomization result. Third, half of the village birth attendants in the region have no formal training, and the other half have inadequate or outdated training [24]. Therefore, there is room for improvement through the proposed maternal education program. Fourth, although the region is not well developed, penetration of mobile phone is high (>70%), rendering an education program relying on health communications by mobile phone feasible.

### Development of the Health Education Tool

On the basis of the WHO education materials, we developed a health education tool with mobile phone text messaging for village health workers and pregnant women alike. The original WHO education materials are written documents with both electronic and paper versions [2]. These documents are comprehensive, with 28 education modules on details regarding various maternity care including safe motherhood, parenthood, concept of risk and appropriateness for prenatal care visits and testing, labor and delivery, postpartum care, and breastfeeding.

Although the WHO education tool is well founded and validated, it is developed in English and is too long and too detailed to be sent by text messaging effectively. To make it a user-friendly, text messaging–based tool acceptable by village health workers and pregnant women in the study region, it needs to be translated into Chinese and to be shortened and modified. First, we formed a multidisciplinary expert panel comprising a maternity care specialist, a midwife, 2 epidemiologists, a psychologist, and a nutrition scientist. The expert panel made decisions on every step of the translation and modification of the WHO education materials. Second, the original English version of WHO education materials was translated into Chinese by 2 researchers independently. Third, the 2 Chinese translation versions were compared with the original English version by the expert panel. The expert panel revised the inconsistent and inaccurate items to reach the final version of the translated Chinese version. The translated Chinese version was then shortened and modified by the expert panel. Finally, the 28 modules of the original WHO educational materials were packaged into 4 periods: first trimester, second trimester, third trimester, and postpartum. For postpartum, materials were packaged for maternal care and baby care separately. For each period, up to 7 text messages with specific educational instructions were included. The main contents of the text messages are displayed in Multimedia Appendix 1 (details available upon request). In addition, a few modifications were made to suit the local culture and lifestyle. For example, with respect to nutrition items, beef and dairy products were replaced with high-protein foods such as pork, fish, chicken, and egg, which are frequently consumed by local people in the region. The whole process followed a previously developed protocol that included rigorous and accurate translations and appropriate appraisal.

### Sample Size

In the original design, we planned to use village as the unit of randomization. The sample size calculation determined that we required 1130 villages (565 villages for each arm) and 10 births per village for the 12 months of the trial (11,300 total births) to achieve 90% power to detect a relative reduction of 30% in the primary outcome (maternal and perinatal death rates) from a control arm rate of 4% using a 2-sided test at the 5% level of significance [22]. These calculations were based on an assumed intraclass correlation of 0.02 for the villages. With a reduction of 30% as the acceptable magnitude of effect for consideration by researchers and/or policy makers, the available study sample is sufficient to answer the study question.

### Recruitment of Study Participants and Randomization

Due to logistical difficulties and budget constraints, we could not use village as the unit of randomization for the cluster trial and we had to use county instead. We, therefore, selected 10 counties in the region and randomly allocated half of the counties to the intervention arm (with text messaging instructions to be sent by county maternal and child health bureaus) and the other half to the control arm (routine care with no text messaging). An independent statistician unrelated to this trial generated the random allocation sequence, and the investigator in charge (RHX) allocated the 10 participating counties to the intervention arm and the control arm accordingly. Village health workers in the 10 participating counties were requested to monitor women of reproductive age who planned to have a baby during the study period, and once a woman was confirmed to be pregnant, she was considered eligible for this study and was recruited into the study by the village health worker. However, any woman who was unable to read or access text messaging through her own phone or her husband’s or family member’s phone was excluded. Recruitment was started on October 1, 2011, and ended on August 31, 2012.

### Delivery of Educational Material

We worked with local mobile phone carriers and maternal and child health bureaus of the intervention counties to install the adapted WHO education material into their wireless telecommunication systems. Text messages containing education materials were delivered to village health workers and pregnant women in the 5 intervention counties according to the pregnancy period recorded by staff at county maternal and child health bureaus.

### Data Collection

Data on mothers’ residence (rural vs urban), gravidity, parity, pregnancy risk status (according to the Chinese national guideline), prenatal visit, prenatal screening, syphilis test, hepatitis B test, folic acid supplementation, mode of delivery, obstetric hemorrhage, maternal death, infant sex, birth weight, perinatal death, thyroid test, phenylketonuria test, and hearing test were collected from study participants by village health workers using the data collection form developed by the research team. Data were collected at the beginning of the diagnosis of pregnancy and in the 42 days postpartum (to meet the definition of maternal death).

### Statistical Analysis

We first compared the distribution of baseline characteristics and then compared maternal and infant outcomes between the 2 arms. Cluster-level analyses proceeded after comparing means and medians of maternal and infant outcomes as proportions for each cluster to ensure that cluster proportions were approximately normally distributed. We then compared mean differences of the maternal and infant outcomes between the 2 arms using a standard unweighted *t* test. Supplementary analysis at the individual level was also performed. In the analysis at the individual level, random effects logistic regression analysis was used. To account for clustering by county, the county was specified as a random effect. To adjust for the small number of clusters, the Kenward-Roger method was used [25]. Odds ratio and 95% CI were expressed as the effect measures, using the control arm (no text messaging) as the reference. The analysis was adjusted for the following baseline characteristics: gravidity, parity, rural residence, household income, high-risk pregnancy status, and infant gender.

## Results

### Participants

**Figure 1:**
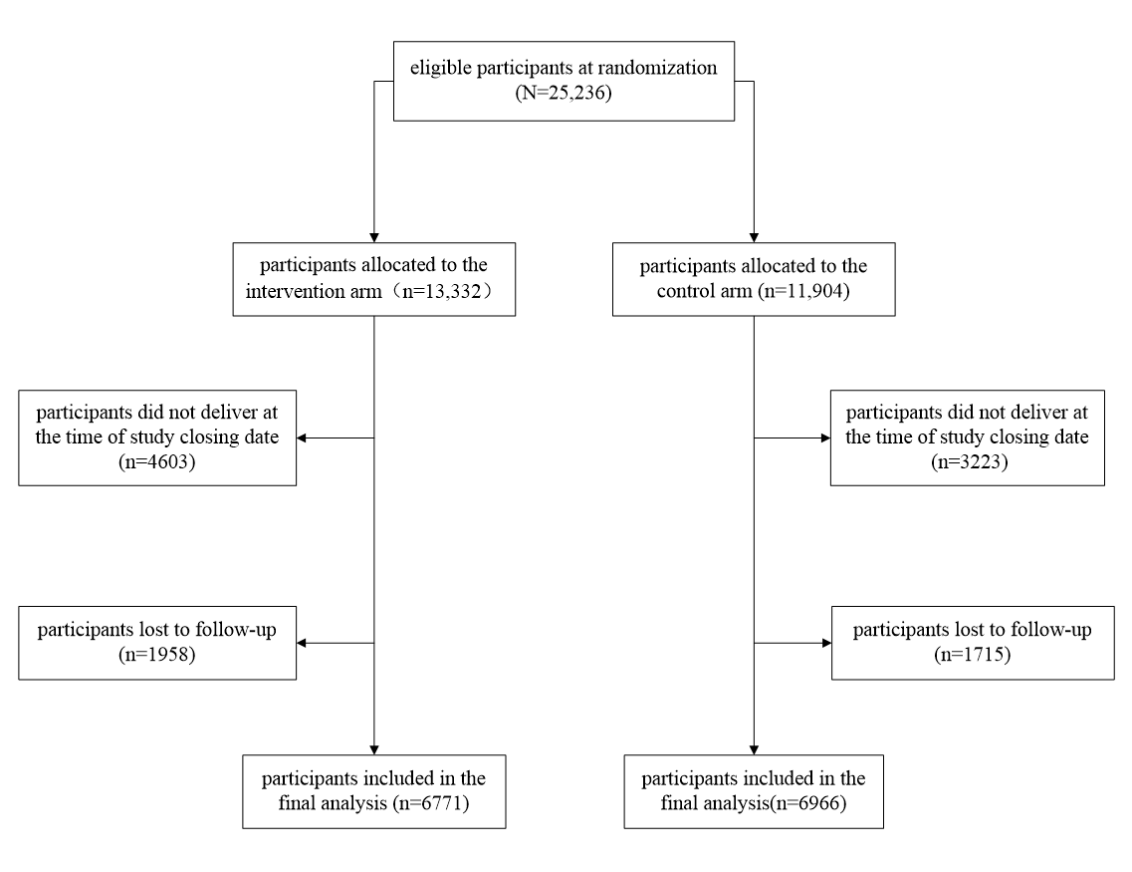
Flowchart of study participants of the text messaging trial in Hunan, China.

Between October 2011 and August 2012, a total of 25,236 pregnant women were recruited into the study (13,332 in the intervention arm and 11,904 in the control arm). Of these, 13,937 (55.2%) women completed the follow-up and were included in the final analysis. Among them, 6771 women were in the intervention arm and 6966 in the control arm. Most of the remaining 11,299 pregnant women excluded from the final analysis did not complete pregnancy before the study closing date (December 31, 2012), rather than being lost to follow-up (Figure 1).

### Baseline Characteristics

Table 1 shows the baseline characteristics of the study population. The study region is a typical rural area of China, with the majority (>90%) of the residents living in rural areas. Maternal and infant baseline characteristics between the 2 arms were generally comparable (Table 1).

As means and medians of county-specific outcome measures were very similar (data available upon request), we did not take log transformations of the data but used *t* tests to compare outcomes between the 2 arms at the county level.

**Table 1:** Comparison of maternal and infant characteristics between the intervention and control arms at the individual level, Hunan, China, 2011-2012.

Characteristics		Intervention arm (N=6771), n (%)	Control arm (N=6966), n (%)
**Gravidity**			
	>1	3285 (48.52)	3478 (49.93)
	1	3486 (51.48)	3488 (50.07)
**Parity**			
	>1	3883 (57.35)	4316 (61.96)
	1	2253 (33.27)	2039 (29.27)
Rural resident		6388 (94.34)	6389 (91.72)
High-risk pregnancy		2488 (36.75)	2841 (40.78)
**Fetal gender**			
	Female	3057 (45.15)	3639 (52.24)
	Male	3684 (54.41)	3304 (47.43)

**Table 2:** Comparison of maternal and infant outcomes between the intervention and control arms at the county level, Hunan, China, 2011-2012.

Outcome	Intervention arm, % mean (SD)	Control arm, % mean (SD)	Percentage mean difference (95% CI)	*P* value (based on *t* test)
Early pregnancy visit	94.8 (2.3)	95.0 (1.1)	0.2 (−5.8 to 6.2)	.94
Prenatal screening	52.5 (12.7)	41.3 (14.3)	−11.2 (−30.9 to 8.5)	.23
Syphilis test	93.2 (7.1)	96.8 (3.3)	3.6 (−4.5 to 11.6)	.34
Hepatitis B test	94.9 (5.4)	98.3 (1.8)	3.4 (−2.4 to 9.3)	.21
Folic acid supplementation	75.6 (13.6)	78.4 (6.3)	2.7 (−12.8 to 18.2)	.69
Cesarean delivery	37.4 (6.8)	42.8 (16.0)	5.4 (−12.5 to 23.3)	.50
Obstetric hemorrhage	0.7 (0.5)	1.2 (0.8)	0.5 (−0.5 to 1.4)	.32
Maternal death	0.0 (0.1)	0.1 (0.2)	0.1 (−0.1 to 0.3)	.47
Perinatal death	1.3 (0.6)	1.5 (0.4)	0.1 (−0.6 to 0.9)	.66
Birth weight <2500 g	3.0 (0.5)	3.7 (2.1)	0.6 (−1.6 to 2.9)	.54
Birth weight >4000 g	1.5 (0.6)	1.6 (0.6)	0.1 (−0.8 to 1)	.80
Thyroid test	86.5 (10.2)	88.1 (5.0)	1.7 (−10.1 to 13.4)	.75
Phenylketonuria test	86.4 (10.2)	88.2 (4.9)	1.8 (−9.9 to 13.5)	.73
Hearing tests	2.3 (0.9)	2.1 (0.9)	−0.2 (−1.5 to 1.1)	.69

**Table 3:** Comparison of maternal and infant outcomes between the intervention and control arms at the individual level, Hunan, China, 2011-2012 (adjusted for gravidity, parity, residence, household income, high-risk pregnancy status, and gender of infant).

Outcomes	Intervention arm (N=6771), n (%)	Control arm (N=6966), n (%)	Crude odds ratio (95% CI)	Adjusted odds ratio (95% CI)
Early pregnancy visit	6308 (93.16)	6644 (95.38)	0.99 (0.98-1.00)	0.99 (0.99-1.00)
Prenatal screening	3291 (48.6)	2381 (34.18)	1.34 (1.28-1.39)	1.25 (1.21-1.31)
Syphilis test	6121 (90.4)	6625 (95.11)	0.98 (0.97-0.98)	0.98 (0.97-0.99)
Hepatitis B test	6229 (92)	6767 (97.14)	0.97 (0.96-0.98)	0.98 (0.97-0.99)
Folic acid supplementation	4733 (69.9)	5431 (77.96)	0.93 (0.91-0.95)	0.92 (0.90-0.93)
Cesarean delivery	2488 (36.75)	2927 (42.02)	0.88 (0.84-0.92)	0.95 (0.91-0.98)
Obstetric hemorrhage	47 (0.69)	77 (1.11)	0.64 (0.45-0.92)	0.42 (0.22-0.80)
Maternal death	3 (0.04)	6 (0.09)	0.52 (0.13-2.10)	—^a^
Perinatal death	84 (1.24)	101 (1.45)	0.96 (0.72-1.27)	0.73 (0.50-1.06)
Birth weight <2500 g	196 (2.9)	210 (3.02)	0.98 (0.81-1.19)	1.20 (0.97-1.47)
Birth weight >4000 g	97 (1.43)	105 (1.51)	0.97 (0.74-1.28)	1.01 (0.76-1.36)
Thyroid test	3977 (58.74)	4156 (59.66)	0.96 (0.95-0.98)	0.96 (0.95-0.98)
Phenylketonuria test	3911 (57.76)	4162 (59.75)	0.96 (0.95-0.98)	0.96 (0.95-0.98)
Hearing test	3935 (58.12)	3989 (57.26)	0.97 (0.95-0.98)	0.97 (0.95-0.99)

^a^Not estimable.

### County Level Comparison

Table 2 compares outcomes between the 2 arms at the county level. Mean (SD) maternal mortality rates were 0.0% (0.1) and 0.1% (0.2), respectively, in the intervention arm and control arm. The corresponding means (SD) for the perinatal mortality rate were 1.3% (0.6) and 1.5% (0.4), respectively, in the intervention arm and control arm. However, these differences were not statistically significant (Table 2).

### Individual Level Comparison

Table 3 displays results of analysis at the individual level. Of the 6771 participants, there were 3 maternal deaths (0.04%) and 84 perinatal deaths (1.24%) in the intervention arm, and of the 6996 participants, there were 6 maternal deaths (0.09%) and 101 perinatal deaths (1.45%) in the control arm. However, the differences were not statistically significant. For secondary outcomes, cesarean delivery (2488/6771, 36.75% cesarean deliveries) and obstetric hemorrhage (47/6771, 0.7% hemorrhage cases) rates were significantly lower in the intervention arm than those in the control arm (2927/6966, 42.02% cesarean deliveries and 77/6966, 1.11% hemorrhage cases), both statistically and clinically (Table 3). No important differences between the 2 arms for other outcomes were observed (Table 3).

## Discussion

### Principal Findings

Our cluster randomized trial in a rural area in Hunan, China, found that it was feasible to deliver maternal education materials by text messaging through a mobile phone to village health workers and pregnant women simultaneously. The results did show some reduction in maternal mortality (3/6771, 0.04%) and perinatal mortality (84/6771, 1.24%) in the intervention arm as compared to the control arm (maternal death rate: 6/6996, 0.09% and perinatal death rate: 101/6996, 1.45%). For secondary outcomes, the rates of cesarean delivery and obstetric hemorrhage were lower in the intervention arm than those in the control arm, both statistically and clinically. This is also expected, as educated women should be better prepared; therefore, the need for cesarean delivery and the incidence of obstetric hemorrhage should be reduced. Although the observed associations between maternal education and maternal and infant outcomes were weak and not demonstrable after taking the cluster effect into consideration, these preliminary results are encouraging and deserve further investigation.

### Strengths and Limitations

To our knowledge, this is the first study that evaluated the impact of WHO’s maternity care education materials for local maternity care education in the remote rural area in China with text messaging. Through mobile phone–based text messaging, we are able to deliver the education materials to a large number of village health workers and pregnant women instantly. The cluster randomized trial is the appropriate design to assess the effect of a maternal education program, as it can be implemented with high efficiency and reduce the chance of contamination [22].

There are several reasons that may explain why our study failed to find an impact of a promising education tool delivered by an efficient method. First, because of implementation difficulties and budgetary constraint, we had to use the county as the unit of randomization. Originally, we planned to use villages as the units of randomization. We realized later that this would have made the trial cost prohibitive: with limited funding, we had to negotiate with local carriers for free text messaging service for this project, which the carriers agreed to only at the county level. There were also logistical considerations: villages lacked the manpower and expertise to deliver education material through text messaging. For these reasons, it was not feasible to use smaller units for randomization. As there were only 10 clusters (counties), we elected to use a more robust cluster-level method of analysis. A disadvantage of analysis at the cluster level is that there could be a loss in power because of data aggregation at the county level. Second, only about half of the recruited women were included in the final analysis. Most of the women were excluded not because they were lost to follow-up but because they had not yet delivered at the time of study termination (again because of budgetary constraints). The loss to follow-up is unlikely to introduce bias because both the intervention and control arms terminated at the same time. However, the substantial loss of study subjects resulted in lower power. Third, because of limited funding, we were not able to vigorously promote, implement, and monitor the maternal education program. For example, we did not track whether or not the village health workers and pregnant women received the text messages, actually read the messages, and if they found the text messages helpful. We did not have the capacity to provide additional assistance to the village health workers and pregnant women if they had difficulties understanding the messages or how to apply them to their own situations. As a result, the program may have not been implemented to the maximum extent possible, thus limiting its impact. Previous studies have suggested that to ensure the success of text messaging–based interventions, efforts should be made to intensively engage with the targeted population [19,20]. Fourth, we have based power calculation on maternal and perinatal mortality rates that were published more than 10 years ago [1]. Maternal and infant health has been improved substantially in the past decade in China, including rural areas [26], which further limited the study power of this trial.

### Implications

Much of the mortality and morbidity in developing countries may be attributable to avoidable risk factors such as unhealthy diets, poor personal hygiene, unsafe delivery by birth attendants, and unintentional injuries; almost all these factors are modifiable [27-29]. For example, postpartum hemorrhage has been identified as one of the most important causes of maternal death in developing countries [27]. On the other hand, evidence generated from clinical investigations, mostly from the industrialized countries, has demonstrated that active management of the third stage of labor can substantially reduce the incidence of severe postpartum hemorrhage [30]. It is, therefore, reasonable to infer that if deliveries in developing countries were managed in the same manner as in industrialized countries, maternal deaths related to postpartum hemorrhage in these countries could be largely prevented. As another example, higher perinatal mortality in developing countries can be attributed in part to the lack of access to high-quality perinatal care for at-risk mothers, fetuses, and newborns [31-33]. Due to the emergent nature of the management of obstetric and neonatal complications and due to the difficulties in transferring at-risk mothers to nearby medical centers in a timely fashion in remote rural areas, instantly accessible information by mobile phone text messaging could provide a helpful tool for village health workers to manage obstetric and neonatal complications locally.

The mobile phone text messaging–based health education tool has been advocated by researchers and health organizations alike [11,12]. The scope and extent of use of this tool with respect to important population health issues have been expanded rapidly, with various trials being designed or launched on repeat suicidal episodes prevention [34], type 2 diabetes prevention [35], detection and management of hypertension in indigenous people [36], diabetes self-management in low- or middle-income countries [37], secondary prevention of coronary heart disease and diabetes [38], and increasing acceptability and use of effective contraception among young women [39]. However, the impact of such a tool in reducing maternal and infant mortality and severe morbidity in low- and middle-income countries such as China has not yet been well documented. The key reason may be lack of rigorous evaluations from randomized trials, and it is crucial to evaluate its beneficial effects using cluster randomized trials.

Although our cluster randomized trial failed to find a statistically significant impact of the maternal education program delivered by text messaging through mobile phone on improved maternal and infant health and health behaviors, the advantages of text messaging in the field of maternal education should not be overlooked. It is able to deliver precisely packaged material to a massive population at a low cost. To send text messages to a massive population through a mobile phone, the senders need to work with local carriers. Therefore, these types of text messages were usually created and distributed by authoritative sources. On the other hand, messages delivered through social media platforms such as Facebook or WeChat, which could be distributed by anyone in the self-established social groups without scrutiny by experts, were often incorrect or even misleading. There is a general agreement that we need rigorous regulations, protocols, and ethical guidelines to correctly apply new technologies (mobile phone apps and text messages included) in the health care environment. Poorly validated information, often created by nonexperts, and a lack of updated data have been mentioned as concerning issues related to health smartphone apps. As such, authors urge different strategies that will provide higher quality evidence for smartphone apps’ effectiveness and contents. This means that nonscientific or not evidence-based information spreading by text messages could be potentially dangerous to patients. Moreover, smartphones are needed to use social media platforms, which are often not affordable for people in remote areas.

### Conclusions

In summary, a cluster randomized trial in a rural area in Hunan, China, suggests that it is possible to deliver maternal education material through text messaging to a massive population at a low cost. Although this exploration trial failed to demonstrate a statistically significant reduction of maternal and perinatal mortality or change in health behavior by maternal education through text messaging, several lessons learned from this exercise could help in the design and execution of future cluster randomized trials evaluating this intervention on maternal and infant health and other health issues. First, the choice of cluster unit for randomization requires balanced consideration. On the one hand, using smaller units such as villages is more efficient in terms of statistical analysis and study power. On the other hand, using larger units such as counties is much easier in the implementation of the trial at a much lower cost. However, using a larger unit of cluster will sacrifice statistical efficiency and study power. Second, to ensure the success of this type of intervention, vigorous promotion, implementation, and monitoring are needed. Finally, refined design considerations such as spreading the text messages after a small face-to-face meeting to explain the goals of text messaging intervention; developing a follow-up source to be sure the women can read and understand the received text messages; using images, videos, or other types of media format that may make the key concepts easier to understand; and assistance for those who may be in need could strengthen the intervention.

## Appendix

Multimedia Appendix 1 The main contents of text messages sent to the health care providers and the women in Hunan, China during the four periods.

Multimedia Appendix 2 CONSORT-EHEALTH checklist (V 1.6.1).

## Abbreviations

mHealth: mobile health
SMS: short message service
WHO: World Health Organization
