# Supplementary material for: The Impact of a Maternal Education Program Through Text Messaging in Rural China: Cluster Randomized Controlled Trial
Source: JMIR Mhealth Uhealth. 2018 Dec 19;6(12):e11213. doi: 10.2196/11213 (PMC6315224; doi:10.2196/11213)
Supplement: Multimedia Appendix 1 [file mhealth_v6i12e11213_app1.pdf]

Multimedia Appendix 1

The main contents of text messages sent to the health care providers and the women during the four periods, Hunan, China

| | 1 | 2 | 3 | 4 | 5 | 6 | 7 |
|---|---|---|---|---|---|---|---|
| First trimester | Healthy lifestyle & environment | Food and nutrition | Folic acid intake | Calculating due date | * | | |
| Second trimester | Antenatal exam (every 4 wks) | Rest and exercise | Food & nutrition | * | | | |
| Third trimester | Antenatal exam (every 2wks) (once a week) | Counting fetal movement | Food & nutrition | Rest and exercise | Personal hygiene | Sex | Childbirth preparation |
| Postpartum<br><br>Mother care | Food and nutrition | Activity & exercises | Sex & family planning | Personal hygiene and environment | Breast care | Check-up | * |
| Baby care | Breastfeeding | Umbilical cord care | Skin care | Diaper changing | Bathing | SIDS preventio n | * |

*Monitoring abnormal signs and symptoms for pregnancy complications
